# Supplementary material for: Complexome profiling of the Chlamydomonas psb28 mutant reveals TEF5 as an early PSII assembly factor
Source: Plant Cell. 2025 Mar 17;37(6):koaf055. doi: 10.1093/plcell/koaf055 (PMC12164590; doi:10.1093/plcell/koaf055)
Supplement: koaf055_Supplementary_Data [file koaf055_supplementary_data.zip › psb28_interactive.zip!/plots/intensity/qualityPlots/peptides/PSAF_Cre09.g412100.t1.2.html]

### Cre09.g412100.t1.2

Trivial name: PSAF  
SeqCoverage [median]: 54.20  
Mass: 24.06 kDa  
  
 

| Sequence | MissedCleavage | Mass [Da] | wtIntensity | muIntensity | Score |
| --- | --- | --- | --- | --- | --- |
| AYAKLEK | 1 | 821.46 | 38364.44 | 41584.42 | 169.37 |
| AYAKLEKK | 2 | 949.56 | 0.00 | 5038.46 | 107.29 |
| EENITVSPR | 0 | 1043.52 | 45217.82 | 28909.97 | 177.30 |
| EIIIDVPLATK | 0 | 1210.72 | 600740.26 | 340826.60 | 177.78 |
| ELKTLEKR | 2 | 1015.60 | 0.00 | 672.36 | 126.41 |
| FANYAK | 0 | 712.35 | 1052215.82 | 986017.10 | 137.20 |
| GEAKPTDKEIIIDVPLATK | 2 | 2037.14 | 1218623.77 | 1225449.86 | 190.83 |
| GTLLEKEENITVSPR | 1 | 1684.90 | 2328191.07 | 2704762.71 | 224.59 |
| LAWQGAGWPLAAVQELQR | 0 | 1993.05 | 109533.89 | 0.00 | 142.39 |
| LAWQGAGWPLAAVQELQRGTLLEKEENITVSPR | 2 | 3659.94 | 2746.01 | 0.00 | 25.31 |
| LKQYEADSAPAVALK | 1 | 1602.86 | 942101.48 | 1161605.07 | 217.88 |
| LKQYEADSAPAVALKATMER | 2 | 2191.13 | 6351.30 | 15996.33 | 83.25 |
| PTDKEIIIDVPLATK | 1 | 1651.94 | 111596.58 | 146181.11 | 122.08 |
| QYEADSAPAVALK | 0 | 1361.68 | 875909.52 | 504768.53 | 336.17 |
| QYEADSAPAVALKATMER | 1 | 1949.95 | 7363.87 | 10197.99 | 101.67 |
| QYLIAVK | 0 | 833.50 | 608354.68 | 458742.01 | 175.93 |
| QYLIAVKGEAKPTDK | 2 | 1659.92 | 26952.78 | 2745.99 | 119.31 |
| RLKQYEADSAPAVALK | 2 | 1758.96 | 1028.52 | 1330.61 | 86.64 |
|  |  |  | 7975291.8 | 7634829.1 |  |
